# Behavioral Responses to Epidemics in an Online Experiment: Using Virtual Diseases to Study Human Behavior

**DOI:** 10.1371/journal.pone.0052814

**Published:** 2013-01-09

**Authors:** Frederick Chen, Amanda Griffith, Allin Cottrell, Yue-Ling Wong

**Affiliations:** 1 Department of Economics, Wake Forest University, Winston-Salem, North Carolina, United State of America; 2 Department of Computer Science, Wake Forest University, Winston-Salem, North Carolina, United State of America; The Queensland Institute of Medical Research, Australia

## Abstract

We report the results of a study we conducted using a simple multiplayer online game that simulates the spread of an infectious disease through a population composed of the players. We use our virtual epidemics game to examine how people respond to epidemics. The analysis shows that people's behavior is responsive to the cost of self-protection, the reported prevalence of disease, and their experiences earlier in the epidemic. Specifically, decreasing the cost of self-protection increases the rate of safe behavior. Higher reported prevalence also raises the likelihood that individuals would engage in self-protection, where the magnitude of this effect depends on how much time has elapsed in the epidemic. Individuals' experiences in terms of how often an infection was acquired when they did not engage in self-protection are another factor that determines whether they will invest in preventive measures later on. All else being equal, individuals who were infected at a higher rate are more likely to engage in self-protective behavior compared to those with a lower rate of infection. Lastly, fixing everything else, people's willingness to engage in safe behavior waxes or wanes over time, depending on the severity of an epidemic: when prevalence is high, people are more likely to adopt self-protective measures as time goes by; when prevalence is low, a ‘self-protection fatigue’ effect sets in whereby individuals are less willing to engage in safe behavior over time.

## Introduction

Thanks to globalization, urbanization, and air travel, infectious diseases can spread faster, wider, and to more people today than ever before [1]. Moreover, diseases such as HIV/AIDS and TB continue to exert a significant burden on the world population, especially the people living in developing countries [2], [3].

Understanding how—and to what extent—people would change their behavior in response to an epidemic is critical for formulating public health policies to control the spread of infectious diseases. This is so since behavioral changes, even when there are no treatments or vaccines available, can be highly effective at slowing or even stopping disease transmission. For instance, a shift in behavior towards safer sexual practices lowered the prevalence of HIV/AIDS considerably in some developing countries [4]–[7]. It has also been argued that behavioral responses, such as wearing of mask, more frequent washing of hands, and avoiding crowded places, of people to the 2003 SARS outbreak contributed significantly to the containment of that epidemic [8].

There are several factors that appear to affect individuals' incentives to adopt precautionary measures in response to an epidemic. Surveys conducted during the 2009 H1N1 pandemic suggest that people's willingness to engage in self-protective behavior depends positively on, among other variables: the level of anxiety regarding the disease, the perceived risk of the disease, the perceived efficacy of the self-protective measures, and household size [9]–[13].

From the perspective of formulating public health policy, it is important to know how changes in individuals' incentive structures affect their level of self-protective behavior. Public policies, such as subsidizing vaccines, recommending social distancing, or providing updates on the severity of an epidemic, in general alter people's decision environments, which in turn can lead to behavior modifications. It is thus crucial for determining the optimal way to contain the spread of infectious diseases that we have a full grasp of how people make decisions regarding self-protective behavior during epidemics.

However, data on how people respond to changes in their incentive structure, such as those resulting from government policies, are not always readily available in the context of infectious disease epidemiology. When an epidemic strikes, it is often simply not feasible to perform controlled experiments that allow us to compare the effects of different policies on a population. This is why epidemiologists rely heavily on the use of mathematical models to predict the effects of various policies and control measures [14]. Of course, the predictions of these models depend critically on the assumptions that the modeler makes about the behavior of people. When forecasting, for instance, the severity of a flu epidemic, the modeler may need to make some assumptions about the fraction of the population that would choose to get a flu shot. When looking at how many people would become infected with a sexually transmitted disease (STD) if certain policies were implemented, the epidemiologist may have to specify, among other things, how many sexual partners people typically have. Thus, unless the models employ the right behavioral specification, their predictions regarding the course of an epidemic or the impact of public policies can be off target.

Some recent advances in the theoretical modeling of infectious disease transmission have incorporated the analytical tools from the fields of economics and game theory to capture more realistically the complex interplay between human behavior and the spread of infectious diseases [15]–[21]. Many of the results and implications derived from these models, however, are ultimately dependent on the assumptions that are made about how people arrive at their decisions when faced with choice problems.

In order to better understand people's willingness to engage in self-protective behavior during an epidemic, we created a simple dynamic multiplayer online game that simulates the spread of an infectious disease through a population composed of the players. In every round of the game, healthy players have the option to choose—at a cost—a protective action that reduces the likelihood of getting infected. Players receive points based on their simulated health status and how often they choose the self-protective action.

The use of our multiplayer virtual epidemics game to study how people respond to an infectious disease offers many advantages, including the ability to alter the players' decision environments and the epidemiological properties of the disease such as its transmission probability and the duration of infection. It also allows us to obtain a wealth of data on the players' behavior by tracking their decisions and the outcomes of their decisions throughout the course of the game. Our virtual epidemics game is similar in spirit to the ‘havatar’—human-avatar pairing—framework that others have proposed recently to study the spread of HIV/AIDS [22]. Methodologically, our study follows a growing trend in economics of utilizing controlled experiments to study economic decision-making (see [23] and [24] for an overview of some major results from the field of experimental economics). To the best of our knowledge, our work is the first virtual experimental study in economic epidemiology.

Here, we report the results of a study we conducted using our epidemics game where the disease has the property that infected individuals recover after a certain amount of time and are once again fully susceptible. This general assumption is appropriate for some real world diseases, in particular STDs such as chlamydia and gonorrhea. We look at the effects of various factors such as prevalence, cost of the protective action, and individuals' infection history to determine what drives people's decision to adopt self-protective measures during an epidemic.

## Materials and Methods

The version of the virtual epidemics game that we used in our study is based on a susceptible-infected-susceptible (SIS) model of infectious disease transmission. We give a general overview of the game in this section; a more detailed description of the game setup is provided in Supporting Information S1. The game has *T* time periods or rounds, and every player is either ‘healthy’ or ‘infected’ in any round. Infection lasts for *τ*<*T* rounds, after which a player becomes healthy. In any round, a healthy player can choose a safe action (self-protect) or a risky action (not self-protect) by clicking on the appropriate button on the computer screen. The safe action guarantees that the player will remain healthy in the following (one) round. As an example, in the context of STDs, the safe action can be thought of as using condoms, which can be highly effective in preventing the spread of disease [25]. If the player chooses the risky action, then the player becomes infected in the next round with a probability that is proportional to the fraction of players that are infected; specifically, the probability that a healthy player in round *t* will get infected in round *t*+1 without taking the safe action is
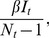
(1)where β is the transmission probability, *I_t_* is the number of infected players in round *t*, and *N_t_* is the total number of players in round *t*. Infected players have no decisions to make. We note that, since players in our study have the option to discontinue participation at any time, the total number of players may not be constant throughout the game.

In every round (except for the first one) before the healthy players make their choices, all the players are told the disease prevalence—the fraction of players that are infected—in the previous round, i.e., at the beginning of round *t*>1, the players are informed of *I_t_*_−1_/*N_t_*_−1_. Throughout the play of the game, all the players are told the values of *τ* and *T*, as well as what the current round is; additionally, in every round, healthy players are informed that the probability of getting infected in the following round without taking the safe action is determined according to rule (1).

Players earn points in every round based on their health status and the action that is chosen. Players receive more points for being healthy than for being infected. We assume that self-protection is a costly activity, so that healthy subjects who choose the safe action receive fewer points than healthy subjects who choose not to self-protect. Specifically, in any round, a healthy player who chooses the risky action receives *P_H_* points; a healthy player who chooses to self-protect receives *P_H_*−*C* points; and an infected player earns *P_I_* points, where *P_H_*>*P_H_*−*C*≥*P_I_*>0. We chose the following parameter values for the present study: *T* = 45, *τ* = 4, *β* = 0.8, *P_H_* = 0.6, *P_I_* = 0.1. For the cost parameter *C*, we chose two different values: 0.35 and 0.45. These choices were made partly for practical considerations (see the Discussion and Conclusion section for an explanation).

Participants for our study were recruited using online classified advertisements. The first page of the website set up for our study is an informed consent page. After clicking on a button on the computer screen consenting to participate in the study and after signing up, the subjects were randomly assigned to the low cost condition (*C* = 0.35) and the high cost condition (*C* = 0.45). Three players in each condition were randomly selected to be infected in round 1 (with duration of infection equal to *τ* = 4), while the rest started the game in the healthy state. We note that, while all the players were told when they signed up for the study that three of them would be randomly picked to be infected to start the game, they were not reminded of this fact at the start of round 1.

For participating in the study, players received an electronic Amazon.com gift card with a value equal to the total number of points that the players earned in the game. At the end of the game, all players were invited via e-mail to complete a brief questionnaire asking for basic demographic information. The players were given an additional $3 in gift card value for completing the questionnaire. The protocol for the study was approved by Wake Forest University's Institutional Review Board.

For each condition, we had 53 players at the start of the game. Because the length of time for each round is fixed, if in any round a healthy player did not submit a choice by the time the round ended, a default choice of the risky action was assigned to the player. All players were informed at the beginning of every round the duration of each round and that the computer program would automatically select the default action—the risky action—for healthy players if they do not enter a choice by the end of any round. (See Figures S1 and S2 for sample screenshots of the game webpage.)

## Results

Basic demographic information collected from the participants in the study is shown in Table S1. The distribution of participants is very similar across the two conditions, indicating successful random assignment by our game program. To control for individual characteristics which may affect the decision to self-protect, our empirical analysis of players' behavior later on makes use of these demographic data.

**Figure 1:**
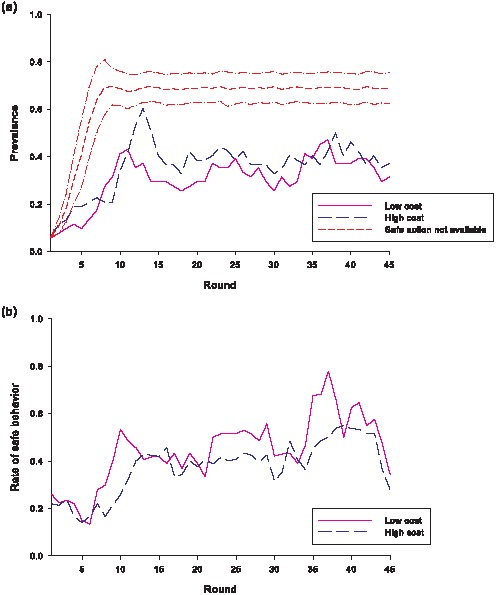
Prevalence of disease and rate of safe behavior in the game. (a) The fraction of players that were infected in each condition. Using computer simulation results, the short dash line shows the mean prevalence over time if no one ever chooses the safe behavior. The computational model used the same parameter values as the virtual epidemics game and had 50 players, with three chosen to be infected in round 1. The simulations were run 500 times, and the two dash-dot lines indicate 1 standard deviation above and below the mean. (b) The fraction of healthy players that chose the safe action in each condition.

Prevalence—the fraction of infected players—as well as the percentage of healthy players using the safe action—which we refer to as the rate of safe behavior—in every round for each of the conditions are shown in Figure 1. An epidemic phase is observed initially—up to rounds 9 or 10. Thereafter, the virtual disease appears to enter into an endemic phase. To avoid issues with multicollinearity, our empirical analysis later on utilizes only observations from round 10 onwards. Therefore, our results can be interpreted as how people behave when a disease is endemic, rather than during the initial stage. As can be seen from Figure 1, a significant proportion of healthy players engaged in self-protective behavior during the course of the game; consequently, the prevalence of the virtual disease was substantially lower than the level that would obtain if no one ever chose the safe action.

To get a sense of how often players were infected, we examined the distribution of players according to the number of times they acquired an infection. The results (see Figure S3) show that the proportion of players who were infected a high number of times (at least three) is greater in the high cost condition, while the low cost condition yielded a higher fraction of players who were infected two or fewer times.

Because choosing the safe action in our game involves actively clicking on a button on the computer screen, while the risky action can be chosen by clicking on the appropriate button or by not clicking on any buttons (recall that the risky action is the default option if a healthy player does not submit an action choice by the end of any round), we also examined the rate with which the players actively entered a choice when they were healthy—which we refer to as the *choice rate* here. The results are given in Table S2. The distribution of choice rates is bimodal in the two conditions, with most of the players having an extremely high or extremely low choice rate. About half of the participants had a choice rate of at least 80%, and 63% of all the players had a choice rate greater than 60%. We note that while 34% of the players had a choice rate of less than 20%, 86% of all the players completed the end-of-study questionnaire. This suggests that some of the players with a low choice rate were in fact attentive to the game and intentionally let the computer select the default option for them during the game. In what follows—unless noted otherwise—we will describe a player as having chosen the risky action whether the player actively entered a choice of the risky action or let the computer pick it as the default option.

**Figure 2:**
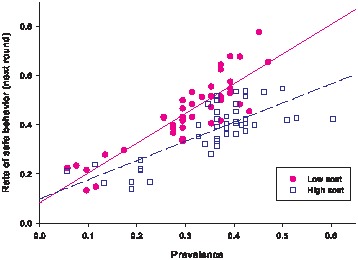
The relationship between prevalence and rate of safe behavior in the following round. The solid and dash lines are the regression lines for the low and high cost conditions, respectively.

We hypothesized that the prevalence of disease in the previous round would affect the propensity a player has for engaging in self-protective behavior. Specifically, the likelihood that a player will choose the safe action should be increasing in the previous-round prevalence since the chances of acquiring an infection from engaging in risky behavior (and, hence, of earning fewer points in the game) are higher when more people are infected. Such prevalence-dependent behavior is predicted by many theoretical models in economic epidemiology, such as those presented in [15]–[17]. As shown in Figure 2, in the high- and low-cost conditions, the fraction of healthy players choosing the safe action in any round rises when the prevalence in the previous round increases. Moreover, the effect of prevalence on behavior is stronger when the cost of the safe action is low. In other words, players' behavior is more sensitive to prevalence when it is cheaper for them to adopt the self-protective action.

**Figure 3:**
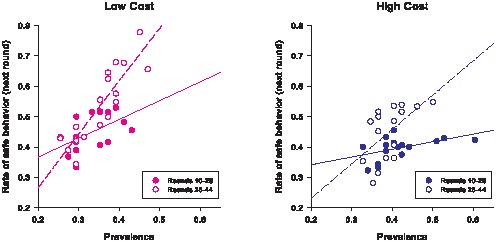
Relationship between prevalence and rate of safe behavior—the effect of the number of rounds elapsed. The solid lines are the regression lines for the data points from rounds 10 to 26; the dashed lines are the regression lines for the data points from rounds 28 to 44.

Interestingly, for either the high- or low-cost condition, the relationship between players' behavior and disease prevalence seems to depend on how many rounds—i.e., how much time—have elapsed in the game. As can be seen from Figure 3, in either condition, players' behavior appears to be more sensitive to a prevalence change in the latter rounds compared to the early-to-middle part of the game.

**Figure 4:**
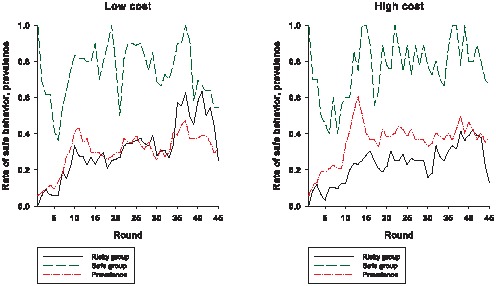
Comparing the rate of safe behavior among those whose first action was the risky action to the rate of safe behavior among those whose first action was the safe action. The “safe” group is composed of those players whose first action was safe. For the low cost condition, there are 13 players in the safe group; for the high cost condition, there are 11 players in the safe group. The “risky” group is composed of those players whose first action was risky. For the low cost condition, there are 38 players in the risky group; for the high cost condition, there are 40 players in the risky group.

A player's risk preferences should affect the player's behavior in the game since the choice between the safe action and the risky action is also a choice between a “sure thing” and a gamble that can yield—relative to the sure thing—either a higher or a lower payoff. Thus, all else being fixed, we would expect the likelihood of choosing the safe action to be higher for someone who dislikes risk immensely compared to someone who is not as averse to risk. Although there is no direct way to measure a player's risk preferences in our study, it is reasonable to assume that players who chose the safe action the first time they had a chance to make a decision are more risk-averse than those who chose the risky option for their first action. (Note that, except for the three players in each condition that were randomly selected to be infected in round 1, a player's first choice of action is the action choice in round 1.) Because a player's first action choice is not a function of the player's experience or history with the virtual epidemic, it should be determined to a large extent by a player's risk preference. Figure 4 shows the comparison between the rate of safe behavior among those who chose the safe action as their first action and the rate of safe behavior among those players whose first action is the risky action. For both conditions, the rate of safe behavior among players who chose the safe action as their first action is higher in every round of the game than the rate of safe behavior among the players who chose the risky action first. When we omit from consideration players who never chose the safe action, and compare the rate of safe behavior of those whose first action was the safe action to the rate of safe behavior among those players who chose the safe action at least once and whose first action was the risky one, we obtain a similar result: except for rounds 39 through 44 in the low cost condition, the rate of safe behavior is higher among the players who chose the safe action for their first action (see Figure S4). These results are consistent with the hypothesis that players who are more averse to risk—and thus more likely to choose the safe action for their first choice—engage in self-protective behavior at a higher rate throughout the virtual epidemic.

To get a better sense of what determines the players' choice of actions in the game when they are healthy, we performed a probit analysis with the probability of choosing the safe action as the dependent variable (we obtained similar results using a logit analysis; see Table S5). We included as independent variables the cost of the safe action, the prevalence of disease in the previous round, and players' first action choice to account for individuals' attitude towards risk. Furthermore, we incorporated the demographic information collected from the end-of-study questionnaire since personal attributes such as gender may affect the propensity a player has for engaging in self-protective behavior.

Because a player's decision in any round may be affected by the player's experiences from taking the risky action from earlier in the game, we also added a measure of how often the player was infected in previous rounds—adjusting for how many times the player chose the risky action in the past—in our empirical model. We refer to this measure by *infectriskratio*, defined to be the number of times a player has been infected divided by the number of times a player's action was the risky one. Given the results shown in Figure 3—specifically, the finding that players' behavior appears to be more sensitive to prevalence later in the game—we included the number of rounds elapsed and a prevalence-round interaction term in the empirical analysis to determine whether having more experience with the virtual epidemic affects the way players respond to the news update on disease prevalence.

Our empirical model is given as follows:
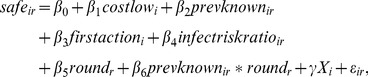
(2)where *safe_ir_* = 1 if the *i*-th individual chooses to self-protect in round *r*, and 0 otherwise. *X_i_* is a vector of demographic characteristics including gender, race, education, marital status, and employment status. Table S4 gives the definitions of the terms in (2). Assuming the error term is normally distributed, we can use a standard probit model to estimate the coefficients in equation (2). As mentioned previously, because prevalence is highly correlated with rounds early in the game—up to round 10—we restricted our data sample to observations from round 10 and beyond.

Marginal effects derived from the probit results are given in Table 1. All players are included in the empirical model in the first two columns, while columns (3) and (4) show the results when restricting the sample to only those players with a choice rate of over 60%. The difference between the first and second columns—and between the last two columns—lies in how the *dependent* variable is defined. In columns (1) and (3), a risky action is considered to be any response other than the safe action—thus, either clicking on the button for the risky action or letting the computer select the default option is counted as a risky action. On the other hand, in columns (2) and (4), only clicking on the button for the risky action is considered to be taking the risky action—not clicking on a button and letting the computer pick the default option is counted as a missing response in the probit analysis. (In defining the independent variables *infectriskratio* and *firstaction*, any response other than the safe action is counted as a risky action.) For brevity, we omitted the results for the demographic variables from Table 1 since, for the most part, the players' behavior does not depend on them (see Table S3). In particular, we note that while some studies of risk-taking behavior have found that women in general are more risk-averse than men [26], we did not find evidence of this in our study as the gender coefficient in our probit analysis is not statistically significant.

**Table 1:** Marginal effects evaluated at the mean using probit results of estimation of equation (2).

	Probability of choosing safe			
	All players		Players with choice rate ≥60%	
	Default choice counted as risky	Default choice counted as missing	Default choice counted as risky	Default choice counted as missing
	(1)	(2)	(3)	(4)
*costlow*	0.104 (0.0975)	0.205^**^ (0.0972)	0.199^*^ (0.105)	0.204^**^ (0.103)
*prevknown*	−0.135 (0.468)	−0.101 (0.518)	−0.212 (0.538)	−0.179 (0.540)
*firstaction*	0.381^***^ (0.0995)	0.183^**^ (0.0836)	0.199^**^ (0.0896)	0.164^*^ (0.0892)
*infectriskratio*	0.227 (0.229)	0.507^**^ (0.200)	0.471^**^ (0.201)	0.534^***^ (0.203)
*round*	−0.0170^**^ (0.00689)	−0.0198^**^ (0.00781)	−0.0192^**^ (0.00785)	−0.0219^***^ (0.00783)
*prevknown×round*	0.0518^***^ (0.0172)	0.0584^***^ (0.0196)	0.0555^***^ (0.0197)	0.0630^***^ (0.0199)
Observations	2296	1560	1641	1521

Except for the first model (column 1)—in which the coefficient on cost is not significant—the probit results show that the likelihood of choosing the safe action is higher when its cost is lower. Specifically, all else equal, players in the low cost treatment are roughly 20 percentage points more likely to choose the safe action than players in the high cost treatment. This indicates that the cost of preventative measures plays a critical role in individuals' decision to engage in self-protection.

Our probit analysis also shows that the players do respond to information regarding disease prevalence—and that their responsiveness to prevalence increases over time. The marginal effects shown in Table 1 indicate that the impact of the reported prevalence on the probability of picking the safe action is strictly increasing by round (from (2), the size of this effect is given by *β*_2_+*β*_6_ *round*). For instance, all else being the same, a 10 percentage point increase in the reported prevalence in round 10 would on average lead to a 4-to-5 percentage point increase in the probability of selecting the safe action. This impact increases to between 9 and 11 percentage points by round 20, and 22 to 23 percentage points by the end of the game.

The variable *firstaction* is statistically significant in all the specifications that we examined, which tells us that players' risk preferences do play a role in how likely they are to engage in self-protective behavior. Specifically, players that revealed themselves to be more risk-averse by choosing the safe action at their first opportunity are roughly 20 percentage points more likely than their less risk-averse peers to choose the safe action in any round, all else equal. This effect is significantly bigger in column (1), at 38 percentage points, but once we focus on players with a high choice rate (at least 60%), we find a fairly robust impact. This indicates that the first result is likely picking up the fact that more risk-averse players are also more likely to have a high choice rate.

Our results show that players' behavior in the virtual epidemic is highly dependent on how many rounds have elapsed in the game and on the outcomes of their (risky) actions in earlier rounds. The coefficient on *infectriskratio* is positive and significant, indicating that players who were infected more often earlier in the game—adjusting for the number of times they chose the risky action—are more likely to self-protect in later rounds. For example, if a player's history increased from getting infected only once per 4 risky actions (*infectriskratio* = 0.25) to being infected once for every 2 risky actions (*infectriskratio* = 0.5), the player's probability of choosing the safe action would increase by 12.5 percentage points, all else being equal. Therefore, holding prevalence constant, one's history with the disease has a significant impact on self-protection behavior; the worse the experiences one has had with the disease, the more likely the player is to self-protect in the future.

How the number of rounds that have taken place since the start of the epidemic affects players' behavior depends on the disease prevalence. From (2), this effect can be measured by *β*_5_+*β*_6_ *prevknown*. Holding all else constant, if the prevalence is high, say, around 0.4 (and remains fixed), then the likelihood that a player would choose to self-protect increases over time. For instance, from the specification shown in column 1 of Table 1, the probability of selecting the safe action rises by 3.7 percentage points for every 10 rounds that go by. Similar results obtain using the other specifications shown in Table 1. This tendency for players to become more “cautious” as the epidemic progresses is even stronger when the prevalence is higher. Assuming that prevalence is fixed at 0.5, for example, the probability of choosing the safe action increases by 9 percentage points for every 10 rounds.

The opposite, however, is true when prevalence is low, i.e., when the number of infected individuals is small, players are less likely to self-protect over time. For a prevalence level of 0.2, the probability of picking the safe action falls between 6 and 10 percentage points every 10 rounds, depending on which specification we look at. The decline is even steeper—between 12 and 16 percentage points—when the prevalence is 0.1. This result suggests that while an epidemic may originally have a strong impact on behavior, the overall salience of the disease may diminish over time when prevalence is low, thus leading to a decrease in self-protection. Such an effect is reminiscent of condom fatigue—the declining use of condom as a preventive measure—in the context of HIV/AIDS prevention.

## Discussion and Conclusion

Here, we have reported on the results of an online experiment that we conducted to examine the incentives that people have for investing in self-protective action during epidemics. The analysis shows that people's behavior is responsive to the cost of self-protection, the reported prevalence of disease, and their experiences earlier in the epidemic. Specifically, decreasing the cost of the self-protective action increases the rate of safe behavior. Higher reported prevalence also raises the likelihood that individuals would engage in self-protection, where the magnitude of this effect depends on how much time has elapsed in the epidemic. Our results show that the effect of a change in reported prevalence on people's behavior is stronger later on in an epidemic compared to the earlier stages. Individuals' experiences in terms of how often an infection was acquired when they did not engage in self-protection are another factor that determines whether they will invest in preventive measures later on. All else being equal, individuals who were infected at a higher rate are more likely to engage in self-protective behavior compared to those with a lower rate of infection. Lastly, fixing everything else, people's willingness to engage in safe behavior waxes or wanes over time, depending on the severity of an epidemic: when prevalence is high, people are more likely to adopt self-protective measures as time goes by; when prevalence is low, a ‘self-protection fatigue’ effect sets in whereby individuals are less willing to engage in safe behavior over time.

Some significant policy implications for controlling the spread of infectious diseases follow directly from our results. Firstly, making preventive measures available during an epidemic—where the cost of these measures are not prohibitive—can be highly effective in reducing the prevalence of disease. Secondly, people respond to incentives: making these preventive measures less costly—through subsidies, for example—encourages more people to engage in safer behavior that reduces the transmission of disease. It is important to note that the costs of self-protective behavior need not be entirely pecuniary. For instance, the cost of using condoms in the prevention of STDs reflects not only the purchase price of these products, but also the ease with which people can find them, the loss of pleasure one may experience from using them, as well as other factors such as cultural beliefs that impact people's willingness to wear them. In the case of influenza, the cost to an individual of getting a flu shot can include—besides the price of a vaccine—all the adverse side effects that the individual believes it to have. Therefore, decreasing the cost of engaging in self-protection does not necessarily entail lowering the amount of money individuals have to give up in exchange for the protective measures.

Individual attributes such as preferences for risk-taking also determine the likelihood that one will choose to adopt preventive measures in response to an epidemic. Given that people can vary widely in terms of their preferences, it follows that any policy that is highly efficacious in inducing safer behavior in one group of people—say, those that are extremely risk-averse—may have smaller effects on other groups. Similarly, individuals can differ significantly in their infection history. Some people, for instance, may rarely get sick during flu season even without getting vaccinated, while others may be more prone to infections without getting a flu shot. If people react differentially to epidemics based on their infection history, then control policies that do not account for this heterogeneity in behavior may end up being less successful in inducing behavior change than anticipated. Thus, with respect to policy effectiveness, it may be preferable to implement a “menu” of policies and interventions that are tailored for different segments of the population than to attempt to formulate “one-size-fits-all” policies that are aimed at all members of a population.

Lastly, because people's behavior in our study is time- and history-dependent, the results suggest that policy interventions should be dynamic, flexible, and adaptable. Given that individuals are not as responsive in their behavior to increases in reported prevalence early on, policies to actively encourage individuals to adopt protective measures in the initial phases of an epidemic could be particularly important in halting or slowing disease spread later on. The finding that, all else being fixed, individuals' propensity to self-protect can change over time depending on how widespread a disease is suggests that intervention efforts may need to be continually revised throughout the course of an epidemic in order to complement or offset changes in people's willingness to engage in self-protective behavior.

Note that since the safe action in our epidemics game is perfectly effective in blocking transmission, eradication of the virtual disease in either condition can be brought about in any round beyond round 4. If all healthy players choose the safe action for at most 4 rounds (which is the length of infection in the game), then eradication is guaranteed to occur, after which all players can receive the maximum payoff (the payoff from being healthy) without having to incur the cost of being infected or the cost of taking the self-protective action. Theoretical models that utilize analytical tools from the fields of economics and game theory predict that, under certain conditions, if a disease will be endemic when no one has access to protective measures, then the disease will also be endemic when protective measures are available, no matter the cost or the efficacy of these protective measures [27]. The fact that the virtual disease in our experiment appeared to settle into an endemic phase in both conditions is consistent with this prediction and underscores how difficult it is for disease eradication to occur under decentralized independent decision-making in the population.

Some aspects of our study design warrant further elaboration. We utilized an SIS model of transmission in our game mainly for two reasons. Firstly, we wanted to examine how an individual's infection history impacts that individual's future decisions to engage in self-protective activities. The second reason is a pragmatic one: to maximize the number of data points we can gather given a fixed number of players, we sought a game structure that would allow players to be actively involved for the entire duration of the game. For example, without some modifications to the game rules or added features, the susceptible-infected (SI) model—in which a player has no possibility of recovery—or the susceptible-infected-removed (SIR) model—in which an infected player can recover after a certain amount of time and subsequently become immune to infection—does not fulfill this criterion as well as an SIS model. With the SI model, no data points can be collected from a player once the player becomes infected. The same is essentially true of the SIR model since recovered players have no incentive to adopt precautionary measures. This is also one of the reasons why in our study we chose for our ‘safe’ action one that is effective for only one round. If, for instance, we had instead chosen a ‘vaccine’ for our safe action that is (perfectly) effective for multiple rounds (e.g., the duration of the game), then we would not have been able to collect any data points from a player once that player chose the safe action.

Relatedly, our choice of parameter values for the study was—as mentioned earlier—motivated partly by practical considerations. We wanted the game to be long so that we can obtain a large number of observations, but we also wanted the length of time needed for study completion to be reasonable (from the participants' perspective). Since only players in the healthy state had to make decisions in our study, we purposely kept the duration of infection 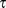 relatively short so that infected players would not be “disengaged” from the game for long. The transmission probability *β* was set high so that the likelihood that the virtual disease would die out before study completion is small, since no useful data can be collected in the disease-free outcome. This is also the reason why we set the number of players infected in round 1 to be three. The choice of how many points to assign to the different health states and of the cost of the safe action was driven by our research budget constraints, the amount of compensation that is typically given to participants of experimental economics studies, and a simple cost-benefit analysis of how the players in our study might behave. We chose the risky action to be the default action since we believe this more accurately reflects what happens in reality. To engage in self-protective behavior such as more frequent washing of hands in the case of flu or using condoms in the context of STDs requires actively and consciously changing one's routine; this is why we set up the game so that one has to manually select the safe action.

Of course, whether one cares about maximizing the number of observations that can be collected per participant or not, our online experimental framework can accommodate different disease transmission processes as well as various types of self-protective actions (including ones that are not perfectly efficacious). We purposely employed a simple random mixing process for our study to better isolate and understand the effects of a few determinants of individual behavior such as reported prevalence, cost of self-protection, and infection history. An important extension of the present study is to consider network effects and how individuals' location in their social network, which determines how likely people will acquire infection from—or transmit infection to—their social contacts, impacts their decision to engage in self-protective behavior during an epidemic.

Another line of research that can be pursued in the future is the formulation of mathematical models of behavior-disease interactions that can account for the results from our study. Such empirically grounded models would provide an analytical framework that can be used to make predictions regarding how, for instance, people would respond to an epidemic under various policies or interventions. While most modeling work in economic epidemiology, following the convention in economics, assumes that agents are rational, forward-looking, payoff- or utility-maximizers, the experimental results presented here suggest that some of these standard assumptions on behavior do not hold in our study. One of the major findings from our study is that one's decision to engage in self-protective behavior is strongly correlated with one's infection history, i.e., people are ‘backward-looking’ in their decision-making process. However, in economic models with forward-looking agents, where all agents are perfectly informed of the ‘rules of the game’, one's past experiences should have no impact on one's current or future behavior. In addition, results obtained from analyses of standard economic models looking at similar decision environments suggest that the players in our virtual epidemics game would utilize a threshold rule in deciding whether to engage in the self-protective behavior or not: choose the safe action if the reported prevalence is above a threshold level; otherwise, choose the risky action. Most of the players in our study, however, did not exhibit such threshold behavior (results not shown). Future modeling work should therefore relax some of the restrictions imposed by the standard economic approach and consider extensions that, for instance, incorporate elements of backward-looking decision-making. Disease transmission models with backward-looking agents have been explored recently (see, for example, [28] and the references therein); these could serve as useful building blocks for formulating models that better account for the experimental results presented here.

Our study design possesses many of the advantages of experiments carried out in controlled laboratory settings, including having a framework to generate data that are otherwise not available and being able to easily manipulate and alter experimental conditions. In addition, since our study takes place entirely online in cyberspace, it has the added benefit of extreme flexibility—as long as one has access to a computer and the internet, experiments can be run anywhere and at any time.

Our setup is also subject to many of the same caveats that apply to laboratory experiments. The game environment abstracts away many aspects of real world infectious diseases. Moreover, it may be difficult to capture all the motivational forces driving people's behavior during real epidemics in an online or virtual setting such as ours. However, as noted by the Nobel Prize-winning economist James Heckman and his colleague, criticizing laboratory experiments in the social sciences as lacking realism misses the point of what “the nature of evidence in science” is; in their words, “(t)he real issue is determining the best way to isolate the causal effect of interest,” and laboratory experiments are no less valid than other methods, such as field experiments and surveys, in accomplishing this [29]. Given that human beings respond to incentives and that understanding incentives is an important component of containing epidemics, furthering our knowledge of what affects people's decision to engage in self-protection—even if obtained from virtual world experiments—can only help us in controlling the spread of infectious diseases.

We note that many of the results from our virtual epidemics study are consistent with those obtained by other researchers using survey-based methods to examine the determinants of self-protective behavior. For instance, a review of previous research on flu shot acceptance finds that demographic variables such as gender, level of education, and ethnicity generally have no impact on vaccination decisions, while perceived likelihood of getting the flu and having had a flu shot previously are significantly correlated with the decision to have a flu shot [30]. Given that the likelihood of infection in our virtual epidemics study is proportional to reported prevalence, our results regarding self-protective behavior with respect to the effects of demographic variables, reported prevalence, and past decision to engage in self-protection precisely mirror those obtained by others looking at flu vaccination behavior. This suggests that an experimental paradigm utilizing virtual diseases can be valuable for studying how people respond to the spread of infectious diseases. Moreover, while surveys can only provide one-time snapshots of people's behavior, our virtual epidemics framework allows researchers to examine behavioral responses in a dynamic context and to analyze how people's incentive to engage in self-protection changes over time. Thus, our experimental framework can serve as a complement to traditional survey methodology to provide a more complete understanding of the determinants of self-protective behavior.

## Supporting Information

Figure S1 **Sample game page for an infected player in the low cost condition.**
(TIF)

Figure S2 **Sample game page for a healthy player in the low cost condition.**
(TIF)

Figure S3 **The distribution of players according to the number of times they were infected in the game.**
(EPS)

Figure S4 **Comparing the rate of safe behavior among those whose first action was the risky action and who chose the safe action at least once to the rate of safe behavior among those whose first action was the safe action.** The “risky” group is composed of those players whose first action was risky and who chose the safe action at least once. For the low cost condition, there are 19 players in the risky group; for the high cost condition, there are 20 players in the risky group.
(EPS)

Table S1 Demographic information for the participants in the study.
(DOCX)

Table S2 The distribution of choice rates in the game. The choice rate *r* is defined to be the percentage of times in which a player actively made a choice on the computer—and did not let the computer choose the default option—when the player's simulated health status was healthy. The numbers do not include the players that dropped out of the study before its completion.
(DOCX)

Table S3 Marginal effects evaluated at the mean using probit results of estimation of equation (2)—complete results.
(DOCX)

Table S4 A full list of the variables in the empirical model (2).
(DOCX)

Table S5 Marginal effects evaluated at the mean using logit results of estimation of equation (2).
(DOCX)

Supporting Information S1 **Detailed description of game set up.**
(DOCX)
