# Supplementary figures and images for: Behavioral Responses to Epidemics in an Online Experiment: Using Virtual Diseases to Study Human Behavior
Source: PLoS One. 2013 Jan 9;8(1):e52814. doi: 10.1371/journal.pone.0052814 (PMC3541346; doi:10.1371/journal.pone.0052814)

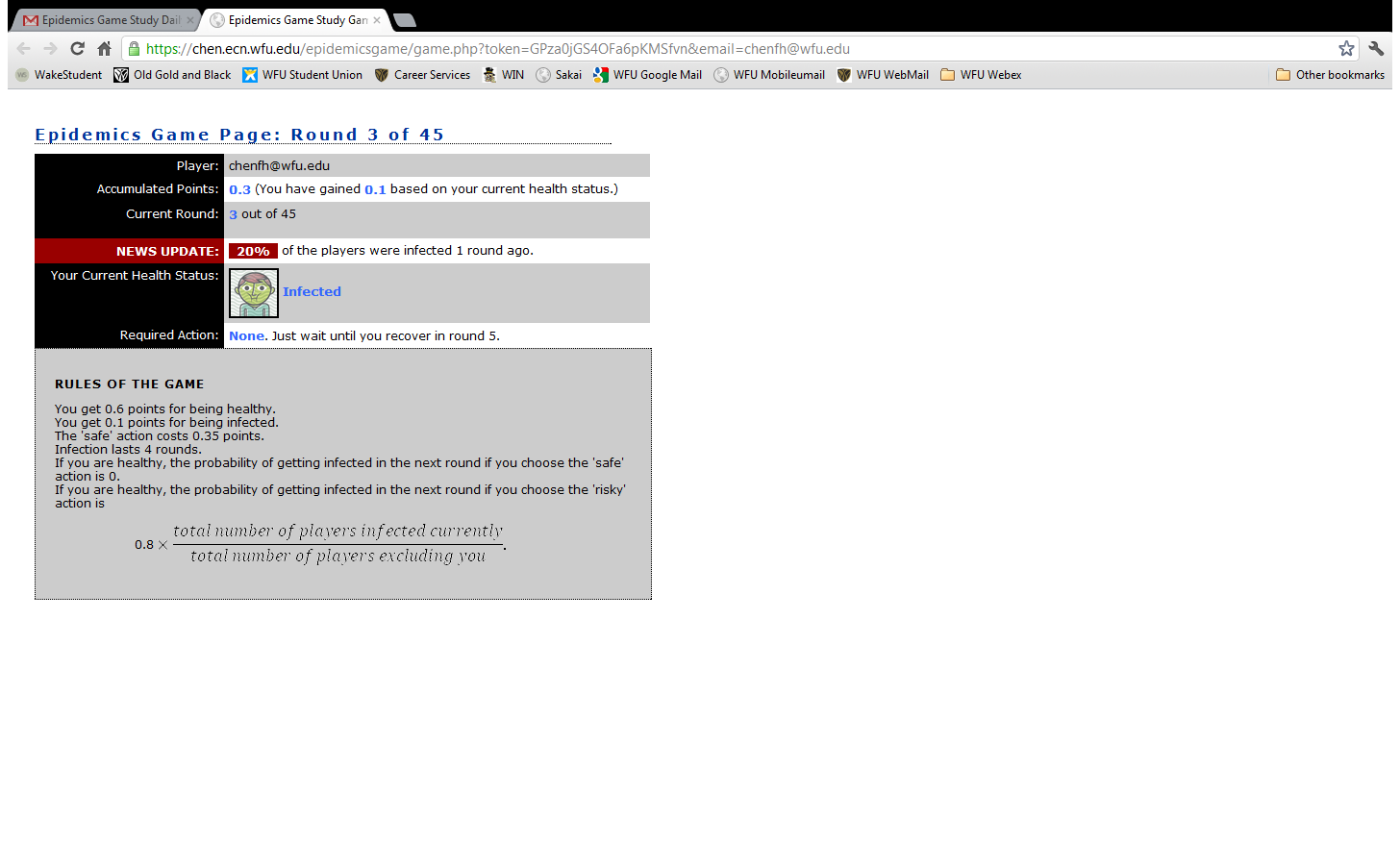

Supplement: Figure S1 — Sample game page for an infected player in the low cost condition. (TIF) [file pone.0052814.s001.tif]

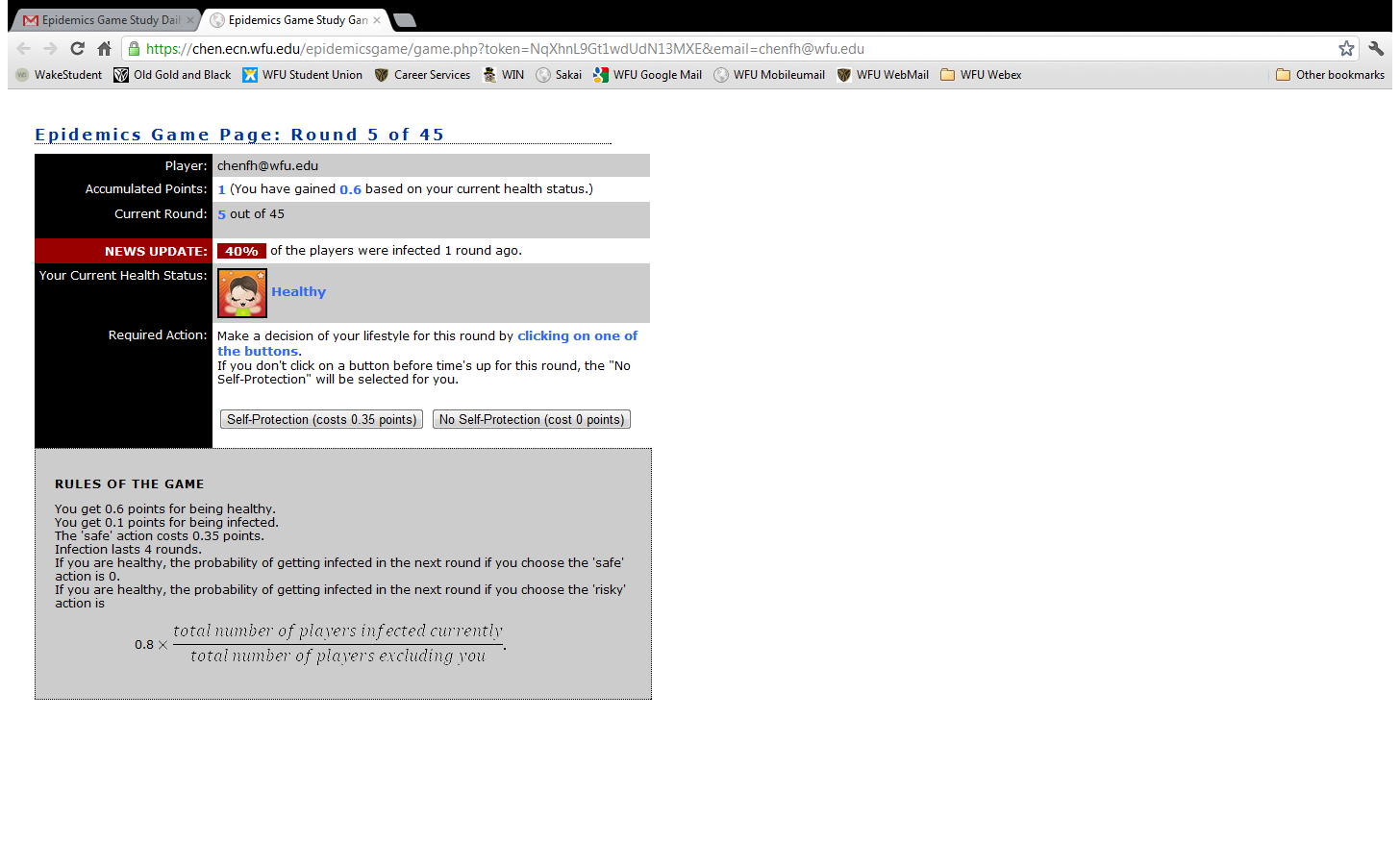

Supplement: Figure S2 — Sample game page for a healthy player in the low cost condition. (TIF) [file pone.0052814.s002.tif]
