# Supplementary material for: Behavioral Responses to Epidemics in an Online Experiment: Using Virtual Diseases to Study Human Behavior
Source: PLoS One. 2013 Jan 9;8(1):e52814. doi: 10.1371/journal.pone.0052814 (PMC3541346; doi:10.1371/journal.pone.0052814)
Supplement: Table S1 — Demographic information for the participants in the study. (DOCX) [file pone.0052814.s005.docx]

|  |  | Low cost condition | High cost condition |
| --- | --- | --- | --- |
| **Number of players** |  | 51 | 51 |
|  | Completed questionnaire | 44 | 44 |
| **Gender** |  |  |  |
|  | Female | 24 | 29 |
|  | Male | 20 | 15 |
| **Age** |  |  |  |
|  | < 25 years | 7 | 12 |
|  | 25 - 44 years | 30 | 28 |
|  | 45 - 64 years | 6 | 4 |
|  | > 65 years | 1 | 0 |
| **Education** |  |  |  |
|  | Some college or less | 21 | 13 |
|  | Bachelor’s degree | 19 | 22 |
|  | Advanced degree | 4 | 9 |
| **Marital Status** |  |  |  |
|  | Married | 21 | 22 |
|  | Never Married | 19 | 21 |
|  | Divorced/Separated | 4 | 1 |
| **Household Income** |  |  |  |
|  | < $50,000 | 20 | 20 |
|  | $50,000 - $99,999 | 14 | 15 |
|  | > $100,000 | 10 | 9 |
| **Employment status** |  |  |  |
|  | Employed | 31 | 28 |
|  | Not Working | 5 | 6 |
|  | Student | 6 | 10 |
| **Race** |  |  |  |
|  | Hispanic | 3 | 3 |
|  | Black/African American | 6 | 4 |
|  | White | 34 | 36 |
|  | Other Race | 5 | 4 |

*Note*: The number of players refers to the players who completed all 45 rounds of the epidemics game. The numbers in the race category for either condition need not sum to the number of players who completed the questionnaire since on the questionnaire there is a question on race as well as a yes/no question on whether one is Spanish/Hispanic/Latino.
