# Supplementary material for: Behavioral Responses to Epidemics in an Online Experiment: Using Virtual Diseases to Study Human Behavior
Source: PLoS One. 2013 Jan 9;8(1):e52814. doi: 10.1371/journal.pone.0052814 (PMC3541346; doi:10.1371/journal.pone.0052814)
Supplement: Table S2 — The distribution of choice rates in the game. The choice rate r is defined to be the percentage of times in which a player actively made a choice on the computer—and did not let the computer choose the default option—when the player's simulated health status was healthy. The numbers do not include the players that dropped out of the study before its completion. (DOCX) [file pone.0052814.s006.docx]

| Choice rate (*r*) | Low cost condition | High cost condition |
| --- | --- | --- |
| *r* ≥ 80% | 25 | 27 |
| 60% ≤  *r* < 80% | 7 | 5 |
| 40% ≤  *r* < 60% | 0 | 1 |
| 20% ≤  *r* < 40% | 0 | 2 |
| *r* < 20% | 19 | 16 |
