# Supplementary material for: Behavioral Responses to Epidemics in an Online Experiment: Using Virtual Diseases to Study Human Behavior
Source: PLoS One. 2013 Jan 9;8(1):e52814. doi: 10.1371/journal.pone.0052814 (PMC3541346; doi:10.1371/journal.pone.0052814)
Supplement: Table S3 — Marginal effects evaluated at the mean using probit results of estimation of equation (2)—complete results. (DOCX) [file pone.0052814.s007.docx]

|  | **Probability of choosing safe** | | | |
| --- | --- | --- | --- | --- |
|  | **All players** | | **Players with choice rate ≥ 60%** | |
|  | Default choice counted as risky | Default choice counted as missing | Default choice counted as risky | Default choice counted as missing |
|  | (1) | (2) | (3) | (4) |
| *costlow* | 0.104 (0.0975) | 0.205^**^ (0.0972) | 0.199^*^ (0.105) | 0.204^**^ (0.103) |
| *prevknown* | -0.135 (0.468) | -0.101 (0.518) | -0.212 (0.538) | -0.179 (0.540) |
| *firstaction* | 0.381^***^ (0.0995) | 0.183^**^ (0.0836) | 0.199^**^ (0.0896) | 0.164^*^ (0.0892) |
| *infectriskratio* | 0.227 (0.229) | 0.507^**^ (0.200) | 0.471^**^ (0.201) | 0.534^***^ (0.203) |
| *round* | -0.0170^**^ (0.00689) | -0.0198^**^ (0.00781) | -0.0192^**^ (0.00785) | -0.0219^***^ (0.00783) |
| *prevknown×round* | 0.0518^***^ (0.0172) | 0.0584^***^ (0.0196) | 0.0555^***^ (0.0197) | 0.0630^***^ (0.0199) |
| *female* | -0.0141 (0.123) | 0.0204 (0.112) | 0.0365 (0.125) | 0.0297 (0.116) |
| *married* | -0.193 (0.124) | -0.0729 (0.120) | -0.0656 (0.119) | -0.0530 (0.119) |
| *black* | 0.142 (0.152) | 0.00778 (0.119) | 0.0306 (0.113) | -0.000676 (0.119) |
| *hispanic* | -0.282^***^ (0.0950) | -0.264^**^ (0.127) | -0.330^**^ (0.147) | -0.273^*^ (0.148) |
| *asian* | -0.157 (0.186) | -0.192 (0.168) | -0.176 (0.165) | -0.181 (0.165) |
| *age* | 0.00285 (0.00423) | 0.00264 (0.00383) | 0.00140 (0.00409) | 0.00230 (0.00395) |
| *badeg* | -0.223^*^ (0.114) | -0.183 (0.118) | -0.154 (0.124) | -0.165 (0.122) |
| *advdeg* | -0.0484 (0.139) | -0.0528 (0.148) | -0.0127 (0.148) | -0.0512 (0.148) |
| *somecoll* | -0.0303 (0.149) | 0.0133 (0.137) | -0.00263 (0.148) | 0.0522 (0.147) |
| *inclt50* | 0.267^**^ (0.131) | 0.0790 (0.141) | 0.0788 (0.158) | 0.0431 (0.149) |
| *inc50100* | 0.306^**^ (0.132) | 0.157 (0.132) | 0.183 (0.153) | 0.137 (0.142) |
| *unemp* | 0.367^**^ (0.180) | 0.177 (0.109) | 0.197 (0.136) | 0.166 (0.107) |
| *selfemp* | 0.235^*^ (0.139) | 0.0873 (0.121) | 0.115 (0.132) | 0.0874 (0.121) |
| *ninworkforce* | 0.0544 (0.107) | -0.0182 (0.101) | -0.0353 (0.100) | -0.0130 (0.101) |
| *demos* | -0.0823 (0.251) | -0.264^***^ (0.0655) | -0.248^*^ (0.137) | -0.253^***^ (0.0655) |
| *setratio0* | 0.435^***^ (0.107) | 0.300^***^ (0.0421) | 0.339^***^ (0.0528) | 0.291^***^ (0.0419) |
| Observations | 2296 | 1560 | 1641 | 1521 |

*Note*: Standard errors in parenthesis (*** significant at 1%; ** significant at 5%; * significant at 10%). The variable *infectriskratio* is set equal to 0 if the player had not yet chosen the risky action.
