# Supplementary material for: Behavioral Responses to Epidemics in an Online Experiment: Using Virtual Diseases to Study Human Behavior
Source: PLoS One. 2013 Jan 9;8(1):e52814. doi: 10.1371/journal.pone.0052814 (PMC3541346; doi:10.1371/journal.pone.0052814)
Supplement: Table S4 — A full list of the variables in the empirical model (2). (DOCX) [file pone.0052814.s008.docx]

| **Term** | **Definition** |
| --- | --- |
| *costlow* | A dummy variable which equals 1 if the player is in the low cost condition, and 0 otherwise |
| *prevknown* | The prevalence in the previous round |
| *firstaction* | A dummy variable which equals 1 if the player’s first action is safe, and 0 otherwise |
| *infectriskratio* | Number of times infected/number of times risky action chosen |
| *round* | Current round number |
| *prevknown* $*$ *round* | Prevalence-round interaction term |
| *female* | A dummy variable which equals 1 if the player is female, and 0 if male |
| *married* | A dummy variable which equals 1 if the player is married, and 0 otherwise (widowed, divorced, separated, never married) |
| *black* | A dummy variable which equals 1 if the player is black, and 0 otherwise (white is the omitted category for the race variables) |
| *hispanic* | A dummy variable which equals 1 if the player is Hispanic, and 0 otherwise |
| *asian* | A dummy variable which equals 1 if the player is Asian, and 0 otherwise |
| *age* | Age of player in years |
| *badeg* | A dummy variable which equals 1 if the player reported that their highest level of education is a four-year degree, and 0 otherwise (high school degree or less is the omitted cateogory for the education variables) |
| *advdeg* | A dummy variable which equals 1 if the player reported that their highest level of education is an advanced degree (PhD, MD, JD, etc.), and 0 otherwise |
| *somecoll* | A dummy variable which equals 1 if the player reported that their highest level of education is some college (but no college degree), and 0 otherwise. |
| *inclt50* | A dummy variable which equals 1 if the player’s household income is less than $50,000, and 0 otherwise (household income greater than $100,000 is the omitted category for the income variables) |
| *inc50100* | A dummy variable which equals 1 if the player’s household income is between $50,000 and $100,000, and 0 otherwise |
| *unemp* | A dummy variable which equals 1 if the player reported being in the work-force but was not currently holding a job, 0 otherwise (employed by outside employer is the omitted category for the employment status variables) |
| *selfemp* | A dummy variable which equals 1 if the player reported that they were self-employed, and 0 otherwise |
| *ninworkforce* | A dummy variable which equals 1 if the player reported that they were not currently in the work-force, and 0 otherwise |
| *demos* | A dummy variable which equals 1 if the player completed the end-of-study questionnaire, and 0 otherwise |
| *setratio0* | A dummy variable which equals 1 if the player had not yet picked the risky action, and 0 otherwise |
| *γ* | A vector of coefficients |
| *ε* | Error term |
