# Supplementary material for: Behavioral Responses to Epidemics in an Online Experiment: Using Virtual Diseases to Study Human Behavior
Source: PLoS One. 2013 Jan 9;8(1):e52814. doi: 10.1371/journal.pone.0052814 (PMC3541346; doi:10.1371/journal.pone.0052814)
Supplement: Table S5 — Marginal effects evaluated at the mean using logit results of estimation of equation (2). (DOCX) [file pone.0052814.s009.docx]

|  | **Probability of choosing safe** | | | |
| --- | --- | --- | --- | --- |
|  | **All players** | | **Players with choice rate ≥ 60%** | |
|  | Default choice counted as risky | Default choice counted as missing | Default choice counted as risky | Default choice counted as missing |
|  | (1) | (2) | (3) | (4) |
| *costlow* | 0.120 (0.108) | 0.203^**^ (0.0983) | 0.210^*^ (0.110) | 0.201^*^ (0.104) |
| *prevknown* | -0.0705 (0.523) | -0.0741 (0.521) | -0.164 (0.558) | -0.153 (0.535) |
| *firstaction* | 0.414^***^ (0.107) | 0.186^**^ (0.0868) | 0.210^**^ (0.0957) | 0.168^*^ (0.0937) |
| *infectriskratio* | 0.218 (0.255) | 0.485^**^ (0.211) | 0.472^**^ (0.222) | 0.507^**^ (0.214) |
| *round* | -0.0170^**^ (0.00777) | -0.0187^**^ (0.00810) | -0.0188^**^ (0.00826) | -0.0206^**^ (0.00804) |
| *prevknown×round* | 0.0529^***^ (0.0192) | 0.0553^***^ (0.0205) | 0.0549^***^ (0.0208) | 0.0597^***^ (0.0205) |
| *female* | -0.0334 (0.146) | 0.0107 (0.116) | 0.0240 (0.132) | 0.0206 (0.119) |
| *married* | -0.229 (0.146) | -0.0904 (0.131) | -0.0833 (0.132) | -0.0684 (0.130) |
| *black* | 0.163 (0.173) | 0.0178 (0.118) | 0.0273 (0.119) | 0.00906 (0.119) |
| *hispanic* | -0.305^***^ (0.101) | -0.285^**^ (0.139) | -0.350^**^ (0.156) | -0.292^*^ (0.162) |
| *asian* | -0.213 (0.212) | -0.217 (0.198) | -0.199 (0.189) | -0.202 (0.192) |
| *age* | 0.00282 (0.00456) | 0.00232 (0.00358) | 0.00137 (0.00399) | 0.00201 (0.00371) |
| *badeg* | -0.231^*^ (0.123) | -0.170 (0.117) | -0.152 (0.126) | -0.152 (0.120) |
| *advdeg* | -0.0466 (0.148) | -0.0470 (0.147) | -0.0135 (0.150) | -0.0457 (0.147) |
| *somecoll* | -0.0295 (0.158) | 0.0101 (0.135) | -0.0115 (0.151) | 0.0431 (0.146) |
| *inclt50* | 0.257^*^ (0.143) | 0.0765 (0.130) | 0.0773 (0.149) | 0.0477 (0.136) |
| *inc50100* | 0.311^**^ (0.142) | 0.151 (0.117) | 0.182 (0.140) | 0.135 (0.125) |
| *unemp* | 0.404^**^ (0.168) | 0.176^*^ (0.0951) | 0.206^*^ (0.123) | 0.164^*^ (0.0942) |
| *selfemp* | 0.219 (0.149) | 0.0646 (0.115) | 0.0937 (0.130) | 0.0652 (0.115) |
| *ninworkforce* | 0.0677 (0.115) | -0.0161 (0.0970) | -0.0329 (0.101) | -0.0130 (0.0970) |
| *demos* | -0.0458 (0.270) | -0.231^***^ (0.0682) | -0.222^*^ (0.127) | -0.222^***^ (0.0674) |
| *setratio0* | 0.455^***^ (0.0996) | 0.285^***^ (0.0467) | 0.328^***^ (0.0544) | 0.276^***^ (0.0463) |
| Observations | 2296 | 1560 | 1641 | 1521 |

*Note*: Standard errors in parenthesis (*** significant at 1%; ** significant at 5%; * significant at 10%). The variable *infectriskratio* is set equal to 0 if the player had not yet chosen the risky action.
