# Supplementary material for: Behavioral Responses to Epidemics in an Online Experiment: Using Virtual Diseases to Study Human Behavior
Source: PLoS One. 2013 Jan 9;8(1):e52814. doi: 10.1371/journal.pone.0052814 (PMC3541346; doi:10.1371/journal.pone.0052814)
Supplement: Supporting Information S1 — Detailed description of game set up. (DOCX) [file pone.0052814.s010.docx]

# Supporting Information

## Game Setup

The online advertisement used to recruit participants includes a link to a webpage set up for the study. To participate, individuals had to give consent by clicking on a button on the webpage and provide a valid e-mail address. If an individual entered an e-mail address or used an IP address that had already been used to sign up for the study earlier, then that individual was not allowed to participate in the study (and was shown a message informing that they cannot sign up for it). Each round of the game lasted 24 hours. On the day the study began, all players received an e-mail informing them that round 1 has started. The e-mail contained a link to the game webpage that allowed players to check their simulated health status, review the rules of the game, and make a choice between the safe and risky action if their health state is healthy. Everyday thereafter till the end of the study, all players were sent an e-mail at 3AM EST informing them that a new round has commenced. All the daily e-mails contained a link to the game webpage where players can view their health status, check the news update on the virtual epidemic, and—for healthy players—choose their action for that round. A link for players to discontinue participation in the study was also included in every e-mail. Figures S1 and S2 show sample screenshots of the game webpage.

## Player Characteristics

Two players from each group withdrew from the study before the end of the game (for the low cost group, the withdrawals occurred immediately following rounds 2 and 7; for the high cost group, the withdrawals occurred immediately following rounds 14 and 43). We did not include in our empirical analysis any of the data from the four players who withdrew. Table S1 shows some demographic information for the study participants. The distribution of choice rates is given in Table S2.

## Additional Results

Figure S3 plots the distribution of players according to the number of times they acquired an infection. How the rate of safe behavior of those whose first action was the safe action compares to the rate of safe behavior among those players who chose the safe action at least once and whose first action was the risky one is shown in Figure S4.

The complete probit results including the effects of the demographic variables are shown in Table S3. Definitions of the terms used are given in Table S4.

All four probit models include the variable *setratio0*. This variable is a dummy variable that distinguishes between the two ways in which *infectriskratio* can be 0. The variable *infectriskratio* is designed to capture a player’s history of infection and risky actions, and is calculated as the ratio of the two. However, for any player who has not chosen the risky action yet, two things will be true. First, the player will have not yet been infected, as it is impossible to become infected if one has chosen only the safe action. Secondly, this player will have an undefined *infectriskratio* (until the round in which the risky action is chosen). To avoid losing these player-round observations in our probit estimations, the variable *infectriskratio* is set to 0 when a player has not yet chosen the risky action. Because *infectriskratio* can equal 0 in two ways—when a player has not chosen the risky action, and when a player has not been infected after having chosen the risky action at least once—the dummy variable *setratio0* is included to differentiate the two cases. The coefficient on *setratio0* is positive and significant in all four models, which is not surprising given that players who have not yet chosen the risky action are probably more likely to continue choosing the safe action than another player that has chosen the risky action already.

For the sake of comparison, the results using logit estimation are given in Table S5.
